# E3 Ubiquitin Ligase NEDD4 Affects Estrogen Receptor α Expression and the Prognosis of Patients with Hormone Receptor-Positive Breast Cancer

**DOI:** 10.3390/cancers15020539

**Published:** 2023-01-16

**Authors:** Yutaka Natori, Junko Suga, Emi Tokuda, Kazunoshin Tachibana, Jun-ichi Imai, Reiko Honma, Yusuke Azami, Masaru Noda, Eisaku Sasaki, Shinya Watanabe, Tohru Ohtake, Shigehira Saji

**Affiliations:** 1 Department of Medical Oncology, Fukushima Medical University, Fukushima 960-1295, Japan; 2 Department of Breast Surgery, Fukushima Medical University, Fukushima 960-1295, Japan; 3 Translational Research Center, Fukushima Medical University, Fukushima 960-1295, Japan; 4 Nippon Gene Co., Ltd., Tokyo 101-0054, Japan

**Keywords:** NEDD4, estrogen receptor, hormone receptor-positive breast cancer, hormone therapy, retrospective cohort study

## Abstract

**Simple Summary:**

Hormone therapy for breast cancer targets estrogen receptors to inhibit the growth of cancer cells. We previously reported that neural precursor cell-expressed developmentally downregulated 4–1 (NEDD4) promotes the degradation of the estrogen receptor α. Therefore, NEDD4 may affect the efficacy of hormone therapy and prognosis in hormone receptor-positive breast cancer patients. The prognosis of patients receiving perioperative neoadjuvant or adjuvant hormone therapy for 5–10 years could be particularly affected by NEDD4. This study revealed that patients with hormone receptor-positive, human epidermal growth factor receptor 2-negative breast cancer with low NEDD4 expression had a good outcome. Additionally, the association of NEDD4 with estrogen receptor α in breast cancer cells was also investigated to reveal the biological mechanisms that influenced clinical results. NEDD4 expression appears to be a predictive factor of the response to hormone therapy.

**Abstract:**

Neural precursor cell-expressed developmentally downregulated 4–1 (NEDD4) is an E3 ligase that leads to the degradation of proteins, including estrogen receptor α. We evaluated whether the expression level of NEDD4 affected the outcome of breast cancer patients. We performed a retrospective cohort study enrolling 143 patients with hormone receptor-positive, human epidermal growth factor receptor 2-negative early breast cancer. Of the 66 patients with high NEDD4 mRNA levels (high NEDD4 group) and 77 patients with low NEDD4 mRNA levels (low NEDD4 group), 98.4% and 96.1%, respectively, of the patients had received neoadjuvant/adjuvant hormone therapy. Disease-free survival and overall survival were significantly longer in the low NEDD4 group than in the high NEDD4 group (*p* = 0.048 and *p* = 0.022, respectively). Western blotting revealed a high expression of estrogen receptor α in the NEDD4-knockdown culture cells. The proliferation of NEDD4-knockdown cells treated with tamoxifen or estradiol deprivation was suppressed, compared with that of NEDD4-expressing cells. Knockdown of NEDD4 in breast cancer cells induced the accumulation of estrogen receptor α and increased sensitivity to hormone therapy. In summary, this mechanism may lead to a better prognosis in hormone receptor-positive breast cancer patients with a low expression of NEDD4.

## 1. Introduction

Neural precursor cell-expressed developmentally downregulated 4–1 (NEDD4) is an E3 ubiquitin ligase that catalyzes the ubiquitination of target proteins, leading to proteolysis of the target proteins in the proteasome [1,2]. NEDD4 targets several proteins involved in cancer growth, including RAS, AKT, and phosphatase and tensin homolog (PTEN) [3,4,5,6,7,8]. In breast cancer cells, NEDD4 promotes human epidermal growth factor receptor (HER)3 degradation, and it has been reported that defects in NEDD4 result in the accumulation of HER3 [9]. With such accumulation of HER3, stimulation of breast cancer cells with neuregulin (NRG), a ligand for HER3, promotes the proliferation of these cancer cells. A defect in NEDD4, resulting in HER3 accumulation, was expected to confer a worse clinical outcome. In contrast, a previous study revealed that a low expression of NEDD4 did not affect the clinical prognosis of patients with breast cancer [10]. Other studies showed that a low expression of NEDD4 was associated with a good prognosis for patients with breast cancer [11,12].

Breast cancer is clinically categorized into three subtypes: HER2-positive breast cancer; hormone receptor (HR)-positive breast cancer; and triple-negative breast cancer [13,14,15]. Each subtype is treated with its own therapy, and each subtype has a different prognosis [16,17,18]. Estradiol (E2) is a ligand for estrogen receptor α (ERα). E2 stimulates ERα, and HR-positive breast cancer cells proliferate via the nuclear translocation of ERα and activation of tyrosine kinase signaling [19]. Hormone therapy targets ERα to inhibit the growth of HR-positive breast cancer cells. We previously reported that NEDD4 promotes the degradation of ERα [20]. NEDD4 accelerates the degradation of ERα and may, therefore, affect the efficacy of hormone therapy and prognosis in HR-positive breast cancer patients.

In this study, we employed clinical and biological approaches to elucidate the prognostic impact of NEDD4 on HR-positive breast cancer. As a clinical approach, we retrospectively evaluated whether the expression level of NEDD4 affected the prognosis of HR-positive breast cancer patients who received neoadjuvant or adjuvant hormone therapy. As a biological approach, the association of NEDD4 with ERα in breast cancer cells was also investigated to reveal the biological mechanisms that influenced clinical results.

## 2. Materials and Methods

### 2.1. Clinical Sample Material

This study was performed in line with the principles of the Declaration of Helsinki and Ethical Guidelines for Medical and Health Research Involving Human Subjects (Ministry of Health, Labour and Welfare in Japan). This study was approved by the Ethics Committee of Fukushima Medical University (approval No. 1953). Informed consent was obtained from all patients.

Breast cancer patients whose expression levels of NEDD4 mRNA were measured at Fukushima Medical University between February 2007 and November 2017 were enrolled in the study. To evaluate the association between HR-positive breast cancer survival and NEDD4, HR-positive HER2-negative breast cancer cases were selected. Patients with pathological stage I and II disease were included in this study. For controls, we also included HR-negative HER2-positive breast cancer and triple-negative breast cancer patients. Clinical information was obtained from medical records in October 2019. Overall survival (OS) was defined as the time from the date of breast cancer diagnosis to the date of death from any cause. Disease-free survival (DFS) was defined as the time from the date of surgery to the date of recurrence or death, whichever occurred first. Patients in whom no event was observed were censored on the day of documentation in their last medical records.

### 2.2. NEDD4 mRNA Expression Analysis

Breast cancer tissues were obtained from surgical or biopsy specimens. The specimens were frozen and processed for total RNA extraction using Isogen (Nippon Gene Co., Ltd., Tokyo, Japan) and for poly(A)+RNA purification using a MicroPoly(A) Purist kit (Ambion, Austin, TX, USA) [21]. The DNA microarray used for poly(A)+RNA was named System 1, where a set of synthetic polynucleotides (80-mers) representing 31,797 species of human transcript sequences, including NEDD4, was printed on a glass slide using a custom array. The DNA microarray used for total RNA was named System 2, in which a set of synthetic polynucleotides (80-mers) representing 14,400 species of human transcript sequences, including NEDD4, was printed on a glass slide using a custom array. For the RNA of the samples, SuperScript II (Invitrogen Life Technologies, Carlsbad, CA, USA) and Cyanine 5-dUTP (Perkin–Elmer Inc., Boston, MA, USA) were used to synthesize labeled cDNA from 2 µg of poly(A)+RNA in System 1 and from 5 µg of total RNA in System 2. Human common reference RNA was prepared by mixing equal amounts of total RNA and poly(A)+RNA extracted from 22 human cancer cell lines (A431, A549, AkI, hBL-100, HeLa, hepG2, hL60, ImR-2, Jurket, k562, kP4, mkN7, Nk-92, Raji, Rd, Saos-2, Sk-N-mC, SW-13, T24, U251, U937, and Y79). With the use of the same method for the human common reference RNA, Cyanine 3-dUTP (Perkin-Elmer Inc.) was used to synthesize labeled cDNA from 2 µg of human universal reference RNA Type I (MicroDiagnostic, Tokyo, Japan) in System 1 and from 5 µg of human universal reference RNA Type II (MicroDiagnostic) in System 2. Hybridization was performed using a labeling and hybridization kit (MicroDiagnostic). Signals were measured using a GenePix 4000 B Scanner (Axon Instruments, Inc., Union City, CA, USA) and converted into primary expression ratios of the cyanine 5 intensity of each specimen to the cyanine 3 intensity of the human universal reference RNA. Each ratio was normalized using GenePix Pro 3.0 software (Axon Instruments, Inc.). The primary expression ratios were converted into log_2_ values, which were designated as the converted values. The converted log_2_ values of the NEDD4 mRNA were used in the present study.

### 2.3. Cell Culture

ERα-positive human breast cancer cell line MCF−7 and human embryonic kidney cell line 293T were purchased from ATCC (Manassas, VA, USA). The ERα-positive human breast cancer cell line T47D was obtained from the Department of Molecular and Functional Dynamics, Graduate School of Medicine (Tohoku University, Sendai, Japan). The cells were cultured in Dulbecco’s modified Eagle medium (DMEM; Fujifilm Wako, Osaka, Japan) supplemented with 10% heat-inactivated fetal bovine serum (FBS; Biowest, Nuaille, France), 100 units/mL penicillin G, and 100 µg/mL streptomycin in a humidified 5% CO2 incubator at 37 °C. As pretreatment for evaluating the cells with E2 stimulation or TAM exposure, the ERα-positive human breast cancer cells were cultured for two days in phenol red-free DMEM (Fujifilm Wako) containing 10% heat-inactivated FBS stripped of steroids using the absorption of dextran-coated charcoal FBS (Biological Industries, Beit HaEmek, Israel). Cells were stimulated with 1 nM E2 (17β-estradiol, Sigma–Aldrich, St. Louis, MO, USA). To simulate clinical tamoxifen therapy, the cells were exposed to 1 nM E2 + 2 µM TAM (4-hydroxytamoxifen, Sigma–Aldrich). The cells were stimulated with 0.4 ng/mL NRG (neuregulin−1β, Bio-Techne, Minneapolis, MN, USA) as a positive control for HER3 activation. The pcDNA3.1 vector containing full-length human NEDD4 was kindly supplied by Dr. Nobuyuki Tanaka (Miyagi Cancer Center Research Institute, Miyagi, Japan). MCF−7 cells with overexpression of NEDD4 were produced by transfection with the vector of NEDD4 using Lipofectamine 2000 (Thermo Fisher Scientific, Waltham, MA, USA).

### 2.4. Small Hairpin RNA (shRNA)-Mediated Knockdown

Vectors of the following shRNA were constructed in the pRSI12-U6-sh-HTS4-UbiC-TagRFP−2A-Puro plasmid (Cellecta, Mountain View, CA, USA): shRNA sequence of CCGGAGAATTATGGGTGTCAA (sh-NEDD4#1), CCGTCAAGTAACTTGGATGTT (sh-NEDD4#2), GCTGAACTATACGGTTCAAAT (sh-NEDD4#3), and no shRNA (sh-control). The sh-NEDD4 or sh-control vector was transfected into 293T cells together with two packaging plasmids, pCMV-VSV-G/RSV-Rev and pCAG-HIVgp (RIKEN Bio-Resource Center, Tsukuba, Japan), using FuGENE HD (Promega, Madison, WI, USA). The 293T cells were used as the packaging cell line to produce lentivirus. At 48 h post-transfection, supernatants were collected and filtered. Supernatants containing lentivirus encoding the shRNA were incubated with MCF−7 and T47D cells for 48 h. The infected cells were selected for additional incubation for 72 h in the presence of 1 µg/mL puromycin (Fujifilm Wako). 

### 2.5. Cell Proliferation Assay

The sh-control or sh-NEDD4-knockdown MCF−7 and T47D cells were seeded in 96-well culture plates (7 × 10^3^ cells/well). After overnight incubation, the medium was replaced with a medium containing ethanol, 1 nM E2, or 1 nM E2 + 2 µM TAM. After incubation, Cell Counting Kit 8 (Dojindo, Kumamoto, Japan) solution was added to each well. After the cells were incubated for 2 h at 37 °C, absorbance was measured using a plate reader (Thermo Fisher Scientific). Absorbance was proportional to cell proliferation.

### 2.6. Western Blots

Western blot samples were prepared from the cultured cells that were lysed in RIPA buffer (40 mM Tris-HCl, pH7.5, 1% NP−40, 150 mM NaCl, 2 mM EDTA, 2 mM Na3VO4, 50 mM NaF) containing protease inhibitor cocktail (Roche, Basel, Switzerland). The samples were then boiled for 5 min at 95 °C. The lysates were centrifuged at 13,000× *g* for 20 min at 4 °C. Proteins in the samples were separated using 4–20% sodium dodecyl sulfate-polyacrylamide gel electrophoresis (Bio-Rad, Hercules, CA, USA) and transferred to polyvinylidene difluoride membranes (Merck Millipore, Burlington, MA, USA). Proteins were blotted using primary and secondary antibodies. The following antibodies were used: NEDD4 #5344S, HER3 #12708S, PTEN #9559S, phospho-HER3 (pHER3 [Y1289]) #4791S, RAS #3965S, ERK1/2 #9102S, phospho-ERK1/2 (pERK1/2 [T202/Y204]) #9101S, AKT #4691T, phospho-AKT (pAKT [S473]) #4060T (the above antibodies were purchased from Cell Signaling Technology, Danvers, MA, USA), ERα #MA5–13304 (Thermo Fisher Scientific), and β-actin #A2228 (Sigma–Aldrich). Protein expression levels were visualized using the SuperSignal West Pico PLUS chemiluminescent substrate (Thermo Fisher Scientific) and ChemiDoc XRS Plus (Bio-Rad). Western blot bands on three independent images were quantified using densitometry (ChemiDoc XRS Plus). The three quantified intensities of bands were normalized by β-actin. The three relative intensities were averaged and made into a graph.

### 2.7. Statistics Analysis

All analyses, including a receiver operating characteristic (ROC) curve analysis, Kaplan–Meier survival analysis, log-rank test, and Student’s *t*-test, were conducted using R version 4.0.3 (R Core Team. R: A language and environment for statistical computing. R Foundation for Statistical Computing, Vienna, Austria. URL https://www.R-project.org/ accessed on 10 November 2020). Kaplan–Meier survival analysis and the log-rank test were used to compare differences in OS or DFS for each clinical categorical variable. Cell proliferation assays were expressed as the mean ± standard deviation. Statistical analysis of the cell proliferation assay was performed using Student’s *t*-test (two-tailed). Statistical significance was set at *p* < 0.05.

## 3. Results

### 3.1. A Low NEDD4 mRNA Level Is Associated with a Favorable Prognosis in HR-Positive Breast Cancer

**Figure 1:**
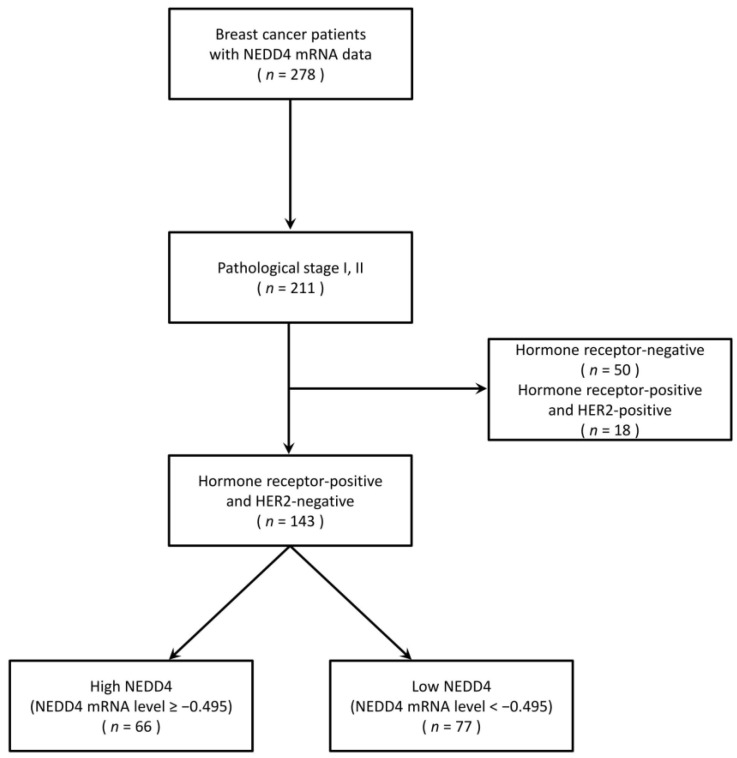
Flow diagram of breast cancer patients whose expression level of NEDD4 mRNA was measured.

**Figure 2:**
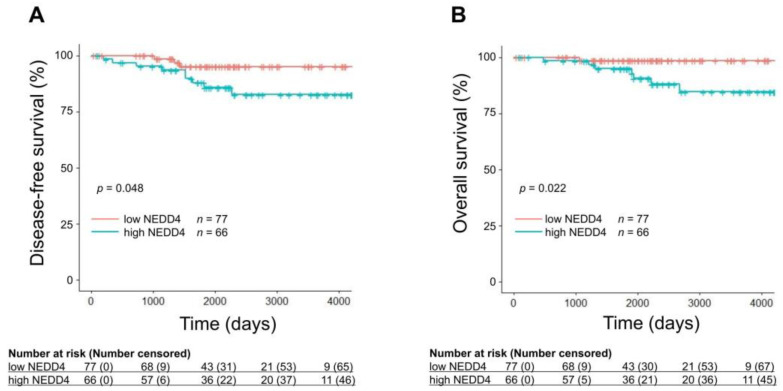
Prognostic impact of NEDD4 on HR-positive HER2-negative breast cancer patients. (**A**) Kaplan–Meier curves for DFS showing a good outcome in the low NEDD4 group (*p* = 0.048). (**B**) Kaplan–Meier curves for OS showing a good outcome in the low NEDD4 group (*p* = 0.022). Log rank (Mantel–Cox) *p*-values < 0.05 were considered statistically significant.

Between February 2007 and November 2017, 278 patients with breast cancer whose NEDD4 mRNA expression levels were measured at Fukushima Medical University were enrolled (Figure 1). Patients with HR-positive HER2-negative breast cancer were selected to evaluate the association between HR-positive breast cancer survival and NEDD4. Patients with pathological stage I or II disease for whom adjuvant hormone therapy had been used for five years or longer were included to investigate the impact of NEDD4 in patients treated primarily with hormone therapy [22,23]. Of these 278 patients, 211 with pathological stage I and II disease and 143 with both HR-positive and HER2-negative breast cancer were selected. As the expression level of the NEDD4 protein in breast cancer cells was reported to be correlated with that of NEDD4 mRNA, the NEDD4 mRNA level was used as an indicator of the NEDD4 protein level in the present study [24]. The NEDD4 mRNA expression levels in cancer tissues were measured. A ROC curve was plotted from the NEDD4 mRNA levels and DFS (Figure S1). The cut-off level at which sensitivity and specificity were maximized was calculated from the ROC curve as −0.495. Of the 143 patients, 66 with NEDD4 mRNA levels ≥ −0.495 were classified into the high NEDD4 group, and 77 with levels < −0.495 were classified into the low NEDD4 group (Table 1). All patients were Japanese women who had undergone breast cancer surgery. The median age at diagnosis was 54 years (range, 30–85 years) and 61 years (range, 29–85 years) in the high NEDD4 and low NEDD4 groups, respectively. The median follow-up period was 6.2 years (range, one month to 12 years). Six patients (4% of the total study population) were followed for less than one year. The proportions of pathological stages in both groups were almost the same: 39.4% stage I and 60.6% stage II in the high NEDD4 group, and 45.5% stage I and 54.5% stage II in the low NEDD4 group. Neoadjuvant/adjuvant hormone therapy was used in 98.4% of the high NEDD4 group and 96.1% of the low NEDD4 group. In the high NEDD4 group, 54.5% received aromatase inhibitors (anastrozole and letrozole) and 43.9% received tamoxifen, compared with 70.1% and 26.0%, respectively, in the low NEDD4 group. Kaplan–Meier survival analysis and the log-rank test were used to compare DFS and OS differences between the high and low NEDD4 groups (Figure 2). The low NEDD4 group had a significantly longer DFS than the high NEDD4 group (*p* = 0.048) and also had significantly longer OS (*p* = 0.022). We also examined the correlation between the expression level of NEDD4 and survival. The group with an extremely high expression of NEDD4 mRNA (≥0.200) had a significantly shorter DFS and OS (Figure S2). According to univariate log-rank analyses, conventional prognostic factors (age, lymph node metastasis, and pathological stage) were not significantly associated with DFS or OS in the study population (Table 2, Figure S3). Therefore, multivariate analysis was not performed. The patients were also divided into three groups based on age (<40, 40–60, and ≥60 years), and no differences in survival were found. These results showed that age (and possibly menopausal status) had no prognostic impact on our study population. Of the 143 patients, 97 were analyzed using scatter plots, and no correlation was observed between the Ki67 and NEDD4 mRNA expression levels (Figure S4).

We also evaluated the prognostic impact of NEDD4 on 50 HR-negative breast cancer patients with pathological stage I and II disease, including HER2-positive or triple-negative cancer. The 50 patients were divided into two groups using NEDD4 mRNA levels ≥ −0.495 (*n* = 12) and <−0.495 (*n* = 38). Kaplan–Meier curves showed that the NEDD4 level was not a prognostic indicator for DFS and OS in the HR-negative breast cancer patients (Figure S5).

### 3.2. NEDD4 Knockdown Increases the ERα Protein Level and E2-Stimulated ERK Signaling

**Figure 3:**
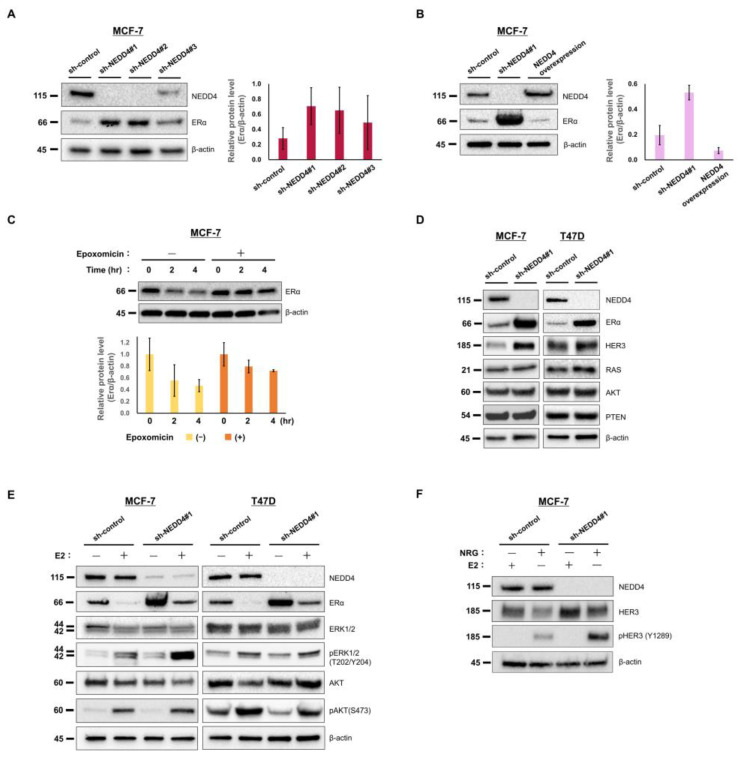
NEDD4 knockdown increased the expression level of ERα and the E2-stimulated phosphorylation of ERK1/2. Whole cell lysates of MCF−7 and T47D cells were subjected to Western blotting. Parts of the full Western blots were cut out and used for the Figure. The original images of the Western blots are shown in the Supplementary Materials: Figure S6–S12 and S14 (**A**) NEDD4 was knocked down with shRNAs with different sequences (sh-NEDD4#1, sh-NEDD4#2, and sh-NEDD4#3). Sh-NEDD4#1 and sh-NEDD4#2 cells showed no expression of NEDD4 and a high expression of ERα. (**B**) MCF−7 cells overexpressing NEDD4 showed a decrease in the expression of ERα. (**C**) Epoxomicin (5 µM) delayed the degradation of ERα in the MCF−7 cell line. (**D**) Expression levels of NEDD4 and proteins associated with NEDD4 in sh-control and sh-NEDD4#1-knockdown cells. Western blotting showed no expression of NEDD4 and a high expression of ERα and HER3 in the sh-NEDD4#1 cells. (**E**) Western blotting of MCF−7 and T47D cells stimulated with 1 nM E2 showed degradation of ERα. Phosphorylation of ERK1/2 (pERK1/2 [T202/Y204]) and AKT (pAKT [S473]) stimulated by 1 nM E2 was observed in the sh-control and sh-NEDD4#1 cells. The phosphorylation of ERK1/2 in the sh-NEDD4#1 MCF−7 cells was greater than that of the sh-control cells. (**F**) Stimulation with 0.4 ng/mL NRG led to the phosphorylation of HER3 (pHER3 [Y1289]), but not with 1 nM E2.

The effect of NEDD4 knockdown on HR-positive breast cancer cells was examined using the ERα-positive breast cancer cell line, MCF−7. NEDD4 was knocked down with shRNAs with different sequences (sh-NEDD4#1, sh-NEDD4#2, and sh-NEDD4#3). According to the Western blots, sh-NEDD4#1 and sh-NEDD4#2 completely knocked down NEDD4, but sh-NEDD4#3 incompletely knocked down NEDD4 (Figure 3A). β-actin was used as an internal control. Western blot analysis of sh-NEDD4#1 and sh-NEDD4#2 cells showed a high expression of ERα compared to cells expressing shRNA for no target gene (sh-control). A little increase in ERα was observed with sh-NEDD4#3 cells. A bar chart based on the quantification of the Western blot bands shows the difference in the ERα expression among these cells.

In MCF−7 cells overexpressing the full length of NEDD4, the ERα expression was decreased, although ERα was not completely degraded (Figure 3B). This result might suggest that the overexpressed NEDD4 was still not enough to degrade all the ERα or that the proteasome degradation system represented the rate-limiting step. Epoxomicin, a proteasome inhibitor, delayed the degradation of ERα in the MCF−7 cell line (Figure 3C). Thus, the involvement of the proteasome system in ERα degradation was demonstrated.

According to these results, we expected that patients with low NEDD4 would show high ERα protein expression. As the Allred score was not available in this data set, we used the percentage of positively ERα-stained cells in clinical specimens. Of the 143 patients, 82 were divided into 2 groups at the NEDD4 mRNA level of −0.495. The percentage of positively ER-stained cells that were evaluated with immunohistochemistry (ER > 50%, 10% < ER ≤ 50%, 1% < ER ≤ 10%, ER ≤ 1%) was compared; however, there was no significant difference in the ER protein expression between the low and high NEDD4 groups (Table S1).

ERα accumulated also in another ERα-positive breast cancer cell line, T47D sh-NEDD4#1 cells (Figure 3D). As previously reported [9], HER3 accumulated in MCF−7 and T47D sh-NEDD4#1 cells. There was no change in the protein level of RAS, AKT, or PTEN. 

ERα is degraded by E2 stimulation in the proteasome system [25]. In the present study, a decrease in ERα expression in MCF−7 and T47D cells stimulated with 1 nM E2 was observed (Figure 3E). Stimulated ERα activates downstream ERK and PI3K/AKT signaling pathways [19]. In sh-NEDD4#1 MCF−7 cells stimulated with 1 nM E2, an increase in ERK1/2 (T202/Y404) phosphorylation was observed; however, AKT (S473) phosphorylation was not increased (Figure 3E). Bar charts based on the quantification of the Western blot bands were added to the Supplementary Materials (Figure S13).

Although HER3 (Y1289) was phosphorylated by NRG, which is a ligand of HER3, HER3 was not phosphorylated by E2 (Figure 3F). E2 stimulation did not activate HER3. HER3 was not involved in the series of results. (The original images of the Western blots in this Section are shown in the Supplementary Materials: Figures S6–S12 and S14).

### 3.3. NEDD4 Knockdown Enhances the Suppression of Cell Growth by Hormone Therapy

**Figure 4:**
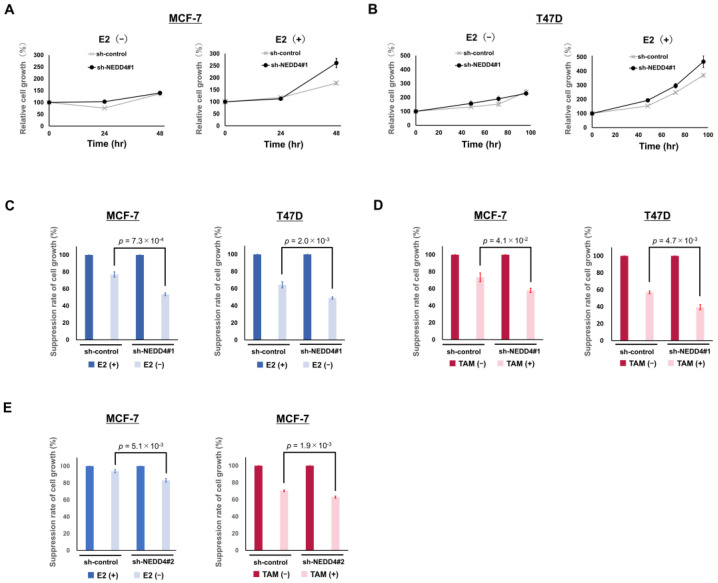
NEDD4 knockdown enhanced E2-dependent cell proliferation and sensitivity to TAM. Cell proliferation was measured using the Cell Counting Kit 8. (**A**,**B**) Comparison of the proliferation of the sh-control and sh-NEDD4#1 cells with or without E2 stimulation. The sh-NEDD4#1 cells of both MCF−7 and T47D cell lines with 1 nM E2 stimulation showed the most rapid cell growth. (**C**) Simulation of the treatment effect on the depletion of E2. In the absence of 1 nM E2, the suppression rate of the MCF7 sh-NEDD4#1 cell growth at 48 h was 53.6% compared with 76.9% for sh-control cell growth. In the T47D cell line, the suppression rate at 96 h was 49.0% (sh-NEDD4#1 cells) compared with 64.4% (sh-control cells). (**D**) Growth of the sh-control and sh-NEDD4#1 cells exposed to 2 µM TAM + 1 nM E2. The suppression rate of the MCF7 sh-NEDD4#1 cell growth with TAM at 72 h was 57.9% compared with 73.3% for sh-control cell growth. In the T47D cell line, the suppression rate at 96 h was 39.4% (sh-NEDD4#1 cells) compared with 57.0% (sh-control cells). (**E**) NEDD4 was knocked down with another shRNA, sh-NEDD4#2. E2 absence or TAM exposure suppressed the cell growth of sh-NEDD4#2 MCF−7 cells more than that of sh-control MCF−7 cells.

Aromatase inhibitors reduce the production of E2 by inhibiting its synthesis in the human body. The effect of E2 reduction by aromatase inhibitors can be mimicked in the absence of E2 stimulation in cultured breast cancer cells. In the sh-control and the sh-NEDD4#1 MCF−7 cells, 1 nM E2 stimulation increased relative cell growth to 177% and 261%, respectively, at 48 h post-stimulation (Figure 4A). However, no difference in cell growth was observed in the absence of E2 stimulation. The same trend was observed in T47D cells (Figure 4B). NEDD4 depletion leads to rapid cell proliferation in response to E2 stimulation. MCF−7 and T47D sh-NEDD4#1 cell proliferation without E2 stimulation at 48 h was suppressed to 53.6% and 49.0%, respectively, of that with E2 stimulation (Figure 4C). Conversely, the sh-control cell proliferation was suppressed to only 76.9% and 64.4%, respectively. The absence of E2 stimulation significantly inhibited the proliferation of the sh-NEDD4#1 cells compared to that of the sh-control cells. 

We also examined the inhibitory effects of TAM on cell proliferation. MCF−7 and T47D sh-NEDD4#1 cell proliferation, when exposed to 1 nM E2 + 2 µM TAM at 72 h, was suppressed to 57.9% and 39.4%, respectively, of that with no exposure (Figure 4D). Conversely, the sh-control cell proliferation was suppressed to only 73.3% and 57.0%, respectively. TAM treatment significantly inhibited cell proliferation in sh-NEDD4#1 cells compared to sh-control cells.

Sh-NEDD4#2 cells in which NEDD4 was knocked down by shRNA with a different sequence from sh-NEDD4#1 also showed the same trend in cell growth as the sh-NEDD4#1 cells under the condition of E2 absence or TAM exposure (Figure 4E). 

## 4. Discussion

NEDD4 is an E3 ubiquitin ligase that ubiquitinates and degrades various proteins in the proteasome [1]. ERα is ubiquitinated by E3 ubiquitin ligases, such as MDM2 and BRCA1, and is degraded in the proteasome [26,27]. In our previous study, we demonstrated that NEDD4 is a novel E3 ligase that is associated with ERα degradation [20]. In this study, we found that the knockdown of NEDD4 led to a high expression of ERα in cultured cells, suggesting that ERα accumulation occurs due to a disorder of the degradation system.

The proliferation of NEDD4-knockdown cells was significantly inhibited in the absence of E2 stimulation compared with that in the control cells. Cell culture in the absence of E2 simulates the depletion of E2 in human cells treated with aromatase inhibitors. Thus, the decrease in the proliferation of NEDD4-knockdown cells in the absence of E2 stimulation suggests that aromatase inhibitors have high efficacy in similar cells in the human body. According to the current therapeutic guidelines, early-stage HR-positive breast cancer patients should receive hormone therapy (aromatase inhibitors or tamoxifen) for approximately 5–10 years [22,23]. In this cohort study, 96.1–98.4% of the patients with HR-positive HER2-negative breast cancer were treated with neoadjuvant/adjuvant hormone therapy (54.5–70.1% aromatase inhibitors, 26.0–43.9% tamoxifen). This cohort study demonstrated that the low NEDD4 group had a significantly longer DFS and OS compared with the high NEDD4 group. Our clinical and biological approaches suggest that ERα accumulation in HR-positive breast cancer with low NEDD4 expression results in significant sensitivity to aromatase inhibitors and prolongs survival. 

A similar pattern was observed in tamoxifen treatment. In the biological simulation of tamoxifen treatment, NEDD4-knockdown cells were more suppressed than NEDD4-expressing cells. In our clinical approach in this study, 26.0–43.9% of the patients were treated with tamoxifen. ERα accumulation caused by low NEDD4 expression may lead to high sensitivity to tamoxifen treatment. In summary, low expression of NEDD4 in HR-positive breast cancer causes the accumulation of ERα, which leads to a favorable response to hormone therapy and prolonged survival. The results of this study suggest that a low expression of NEDD4 is a predictive marker for the response to hormone therapy. 

In clinically obtained specimens, we could not show a correlation between NEDD4 mRNA expression and the percentage of positively ER-stained cells. As approximately 93% of the patients were categorized as having 50% or more ER-positive stained cells, conventional immunohistochemistry evaluation could not detect a difference in the expression levels of ERα accompanied by NEDD4 expression levels.

Another research group reported that the prognosis of HR-positive breast cancer was independent of the expression level of NEDD4, while the prognosis of HR-negative breast cancer was dependent on that level [12]. The prognoses were compared using the web-based public database of the Kaplan–Meier plotter. In the database, patient characteristics, including hormone therapy and the cut-off value of NEDD4 level, were unclear. Although NEDD4 has many other degradation substrates that affect cell growth, this previous study did not address this point. The expression levels of RAS, AKT, PTEN, and HER3 have been reported to be increased by NEDD4 knockdown [1,3,4,5]. However, contrary reports showed that NEDD4 is not involved in PTEN degradation in breast cancer and mouse embryonic fibroblast cells [24,28]. NEDD4-mediated ubiquitination regulates the levels of phosphorylated AKT, but not of total AKT, in MCF−7 and endometrial cancer cells [4,29,30]. As the degradation of RAS via the NEDD4 ubiquitination system is regulated by negative feedback regulation induced by RAS signaling itself, there is no change in degradation without RAS signaling in kidney cells [3]. Thus, the expression levels of RAS, AKT, and PTEN in NEDD4-knockdown cells have been controversial among cancer cell types. In this study, the expressions of RAS, AKT, and PTEN did not change. We believe that the high sensitivity to hormone therapy is independent of RAS, AKT, and PTEN because there was no change in the expression levels of these proteins in NEDD4-knockdown cells. Although NEDD4 knockdown caused HER3 accumulation in our study, it was insufficient to activate HER3. This result is consistent with that of a previous report suggesting that HER3 accumulation does not accelerate cell growth in the absence of a ligand [9]. E2 stimulation did not activate HER3, and the observed cell signaling was independent of HER3.

As a limitation of this study, we did not prove that the ubiquitination of ERα is mediated by NEDD4, followed by the degradation of ERα in proteasomes, in our experiments. Although we demonstrated an inverse correlation between NEDD4 and ERα levels, further studies are required to elucidate the interaction between these two proteins. Univariate analysis showed no difference in survival based on age or lymph node metastasis. Our study population had a relatively good outcome with two-thirds of the patients receiving hormone therapy alone as adjuvant therapy, with a relatively short observation period of 6.2 years. This might be one of the reasons why the analyses showed no difference in survival based on the conventional prognostic factors. As this retrospective cohort study had group biases, including age and hormone therapy drugs, a prospective study is required for statistical accuracy in the future. 

## 5. Conclusions

This study is the first to demonstrate that NEDD4 affects the expression of ERα and influences the prognosis of breast cancer patients. Here, we showed that high ERα expression in HR-positive breast cancer cells via NEDD4 knockdown leads to a high sensitivity to hormone therapy. In a retrospective cohort study, patients with HR-positive HER2-negative breast cancer with low NEDD4 levels had a good outcome. NEDD4 expression seems to be a predictive factor of the response to hormone therapy.

## Figures and Tables

**Table 1:** Clinicopathological characteristics of the patients with HR-positive HER2-negative breast cancer, classified into high and low NEDD4 mRNA groups.

Characteristics	High NEDD4 (*n* = 66)	Low NEDD4 (*n* = 77)
Age (years) median (range)	54 (30–85)	61 (29–85)
Female (%)	66 (100)	77 (100)
Stage (%)		
I	26 (39.4)	35 (45.5)
II	40 (60.6)	42 (54.5)
Lymph node metastasis (%)	23 (34.8)	26 (33.8)
Estrogen receptor (+) (%)	66 (100)	77 (100)
Progesterone receptor (+) (%)	47 (71.2)	67 (87.0)
HER2 (+) (%)	0 (0)	0 (0)
Surgery (%)	66 (100)	77 (100)
Chemotherapy (%)	27 (40.9)	21 (27.3)
Neoadjuvant	2 (3.0)	3 (3.9)
Adjuvant	25 (37.9)	18 (23.4)
Radiotherapy (%)	41 (62.1)	37 (48.1)
Neoadjuvant/adjuvant hormone therapy (%)	65 (98.4)	74 (96.1)
Aromatase inhibitors	36 (54.5)	54 (70.1)
Tamoxifen	29 (43.9)	20 (26.0)
NEDD4 mRNA (range)	−0.258 (−0.494 to –1.105)	−0.775 (−1.681 to −0.496)

**Table 2:** Univariate log-rank analyses for prognostic factors and the NEDD4 mRNA level of HR-positive HER2-negative breast cancer; *p*-values that are statistically significant (*p* < 0.05) are marked with an asterisk (*).

	*p*-Value (Univariate Analysis)	
	DFS	OS
Age ≥ 50 vs. <50 (years)	0.862	0.643
Age ≥ 60 vs. <40 (years)	0.724	0.567
Lymph node metastasis (+) vs. (−)	0.609	0.959
Stage I vs. II	0.062	0.779
Chemotherapy (+) vs. (−)	0.628	0.949
Radiotherapy (+) vs. (−)	0.991	0.580
High NEDD4 vs. Low NEDD4	0.048 *	0.022 *

## Data Availability

The datasets used and/or analyzed during the current study are available from the corresponding author on reasonable request.

## Supplementary Materials

The following supporting information can be downloaded at https://www.mdpi.com/article/10.3390/cancers15020539/s1, Figure S1: ROC curve plotted from the NEDD4 mRNA levels and DFS; Figure S2: Prognostic impact of extremely high NEDD4 mRNA levels; Figure S3: Prognostic impact of conventional prognostic factors; Figure S4: Scatter plot for Ki67 and NEDD4 mRNA expression levels; Figure S5: Prognostic impact of NEDD4 on HR-negative breast cancer patients; Figure S6: Full Western blots of the MCF−7 cell line for Figure 3A; Figure S7: Full Western blots of the MCF−7 cell line for Figure 3B; Figure S8: Full Western blots of the MCF−7 cell line for Figure 3C; Figure S9: Full Western blots of the MCF−7 cell line for Figure 3D; Figure S10: Full Western blots of the T47D cell line for Figure 3D; Figure S11: Full Western blots of the MCF−7 cell line for Figure 3E; Figure S12: Full Western blots of the T47D cell line for Figure 3E; Figure S13: Bar charts based on the quantification of the Western blot bands of Figure 3E; Figure S14: Full Western blots of the MCF−7 cell line for Figure 3F; Table S1: The correlation between NEDD4 and ER expression level in clinical specimens.
