# Supplementary material for: E3 Ubiquitin Ligase NEDD4 Affects Estrogen Receptor α Expression and the Prognosis of Patients with Hormone Receptor-Positive Breast Cancer
Source: Cancers (Basel). 2023 Jan 16;15(2):539. doi: 10.3390/cancers15020539 (PMC9857178; doi:10.3390/cancers15020539)
Supplement: Supplementary file 1 [file cancers-15-00539-s001.zip › cancers-2073665-supplementary.pdf]

Figure S1. ROC curve plotted from the NEDD4 mRNA levels and DFS. The ROC curve for evaluating an optimal cut-off value of NEDD4 mRNA level of hormone receptor-positive HER2-negative breast cancer patients to divide the patients between high and low NEDD4 levels. The cut-off value of the mRNA level was calculated as -0.495 from the ROC curve.

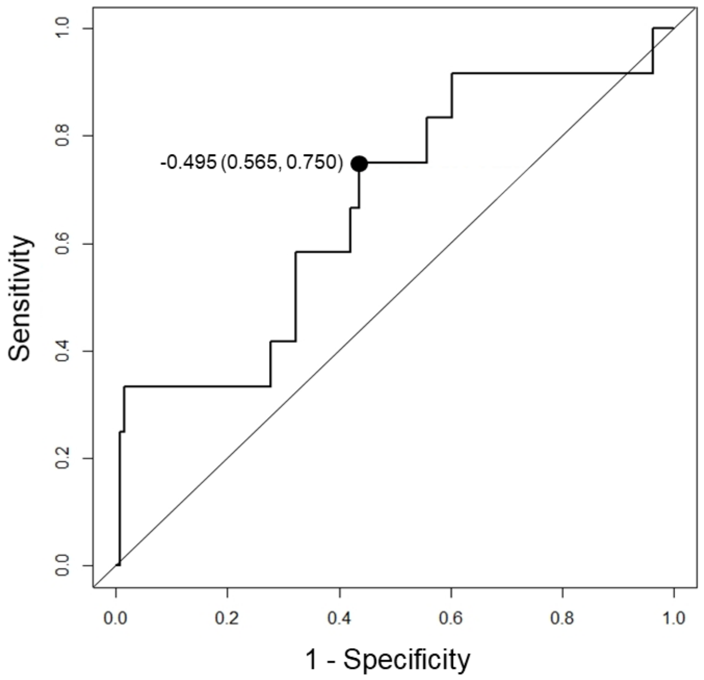

Figure S2. Prognostic impact of extremely high NEDD4 mRNA level on hormone receptor-positive HER2-positive breast cancer patients (≥ 0.200, n = 9 vs < 0.200, n = 134). (A) Kaplan-Meier curves showing very short DFS in those with extremely high NEDD4 mRNA level. (B) Kaplan-Meier curves showing very short OS in those with extremely high NEDD4 level.

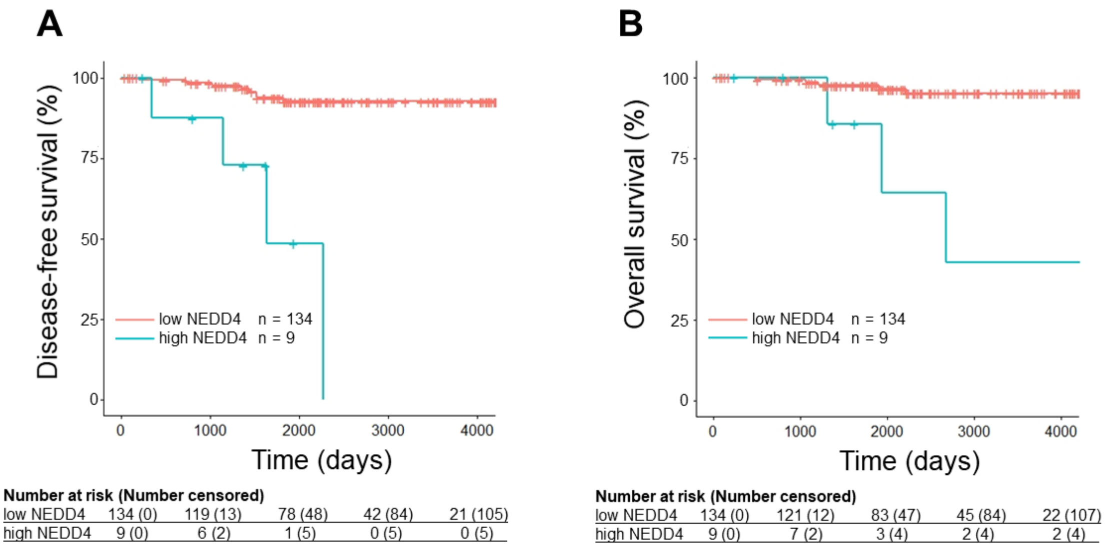

Figure S3. Prognostic impact of conventional prognostic factors (age, pathological stage, lymph node metastasis), use of adjuvant radiotherapy and chemotherapy. (A, B) Kaplan-Meier curves for DFS and OS according to age (< 50 and ≥ 50 years). (C, D) Kaplan-Meier curves for DFS and OS according to age (< 40, 40–60, and ≥ 60 years). (E, F) Kaplan-Meier curves for DFS and OS according to stage I and II. (G, H) Kaplan-Meier curves for DFS and OS according to lymph node metastasis. (I, J) Kaplan-Meier curves for DFS and OS according to use of adjuvant radiotherapy. (K, L) Kaplan-Meier curves for DFS and OS according to use of adjuvant chemotherapy. *P*-values < 0.05 were considered statistically significant.

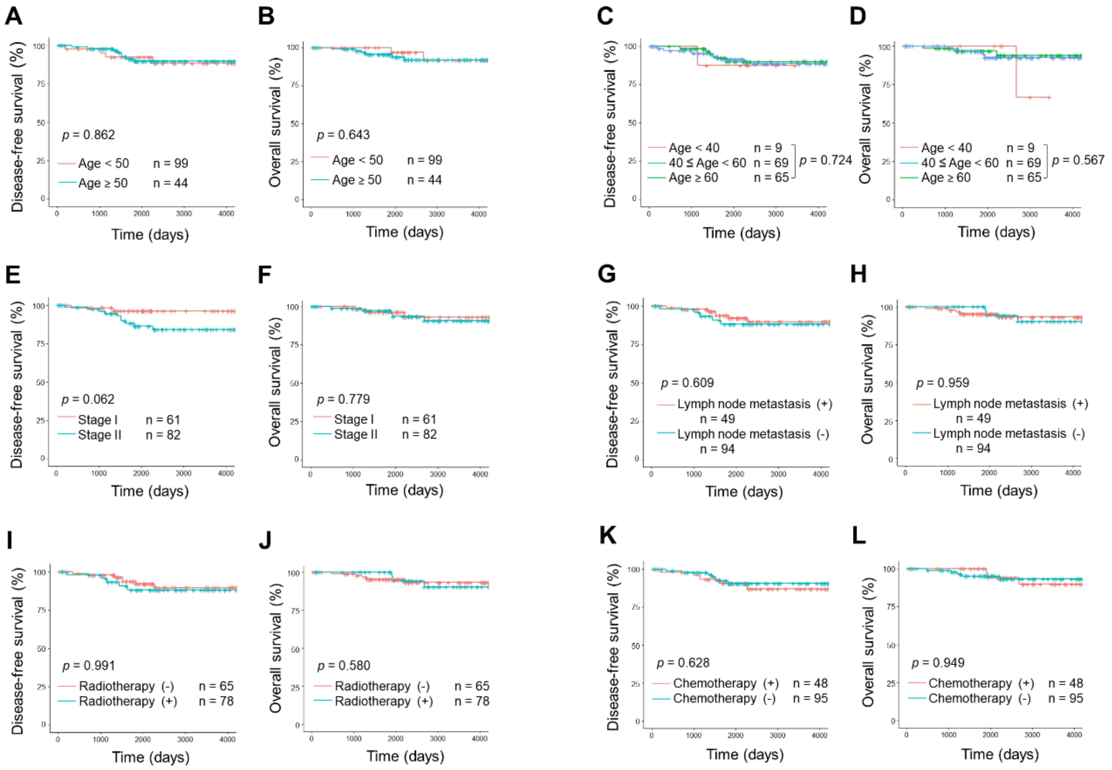

Figure S4. Scatter plot for Ki67 and NEDD4 mRNA expression levels. Of the 143 patients, 97 were analyzed using scatter plots. No correlation was observed between Ki67 and NEDD4 mRNA expression levels.

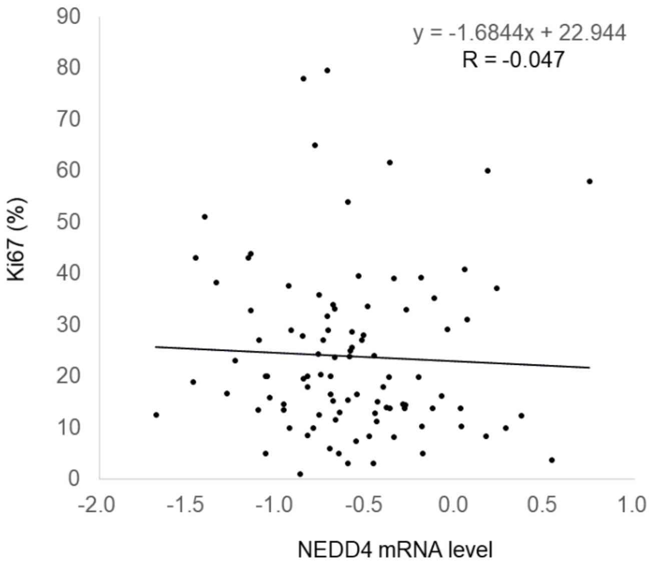

Figure S5. Prognostic impact of NEDD4 on hormone-receptor negative breast cancer patients. The patients with pathological stage I and II including HER2-positive or triple-negative cancer. Of the 50 patients, 12 had NEDD4 mRNA level ≥ -0.495 (high NEDD4 group) and 38 had < -0.495 (low NEDD4 group). (A) Kaplan-Meier curves showing that NEDD4 level was not a prognostic indicator for DFS (p = 0.388). (B) Kaplan-Meier curves showing that NEDD4 level was also not a prognostic indicator for OS (p = 0.420). Log rank (Mantel-Cox) p-values are indicated. P-values < 0.05 were considered statistically significant.

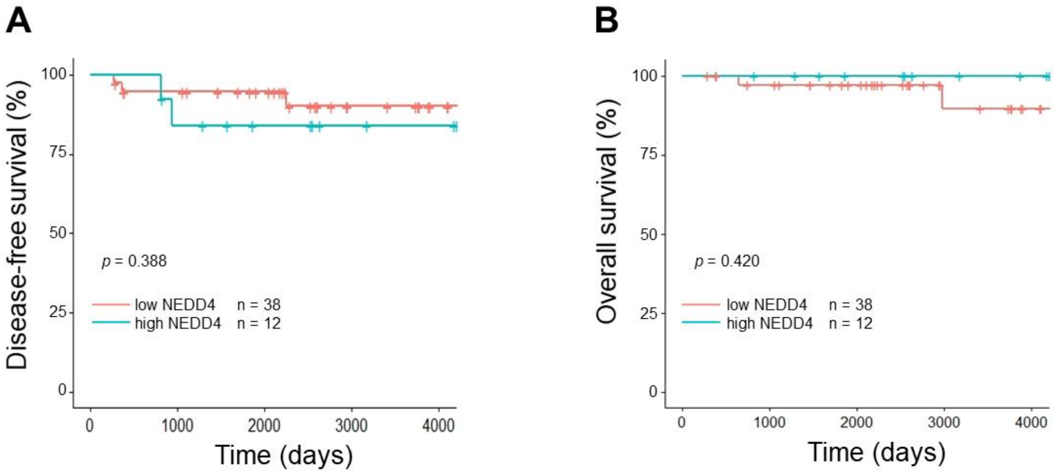

Number at risk (Number censored)

low NEDD4	38 (0)	33 (3)	26 (10)	12 (23)	6 (29)
high NEDD4	12 (0)	9 (1)	5 (5)	3 (7)	2 (8)

Number at risk (Number censored)

low NEDD4	38 (0)	33 (4)	26 (11)	12 (24)	6 (30)
high NEDD4	12 (0)	11 (1)	6 (6)	4 (8)	2 (10)

Figure S6. Full western blots of MCF-7 cell line for Figure 3A. Fields in the dotted line were cut out and used for the figure. Images of the same membrane that was re-blotted with different antibodies were grouped.

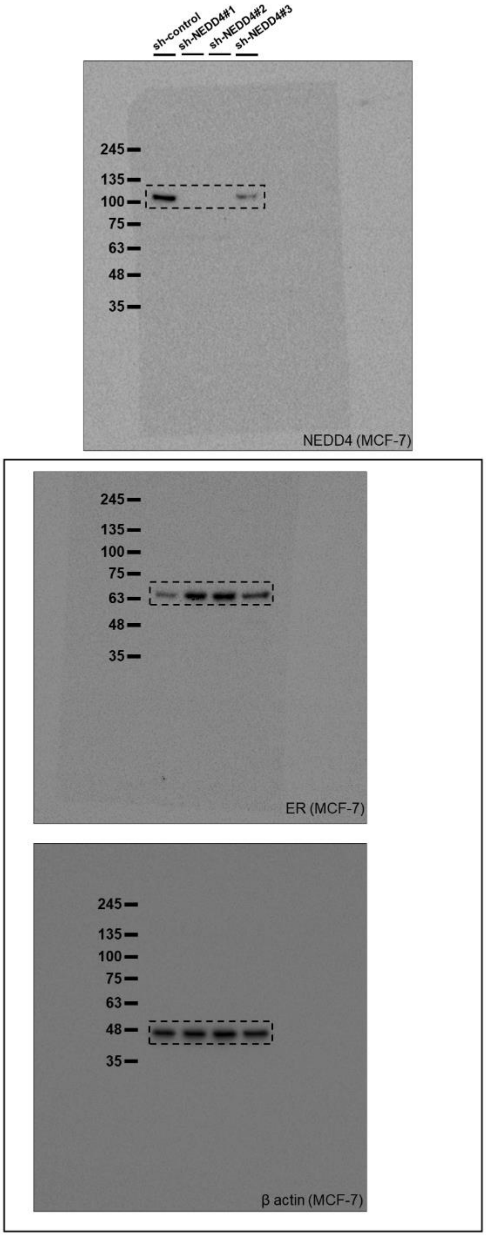

Figure S7. Full western blots of MCF-7 cell line for Figure 3B. Fields in the dotted line were cut out and used for the figure. Images of the same membrane that was re-blotted with different antibodies were grouped.

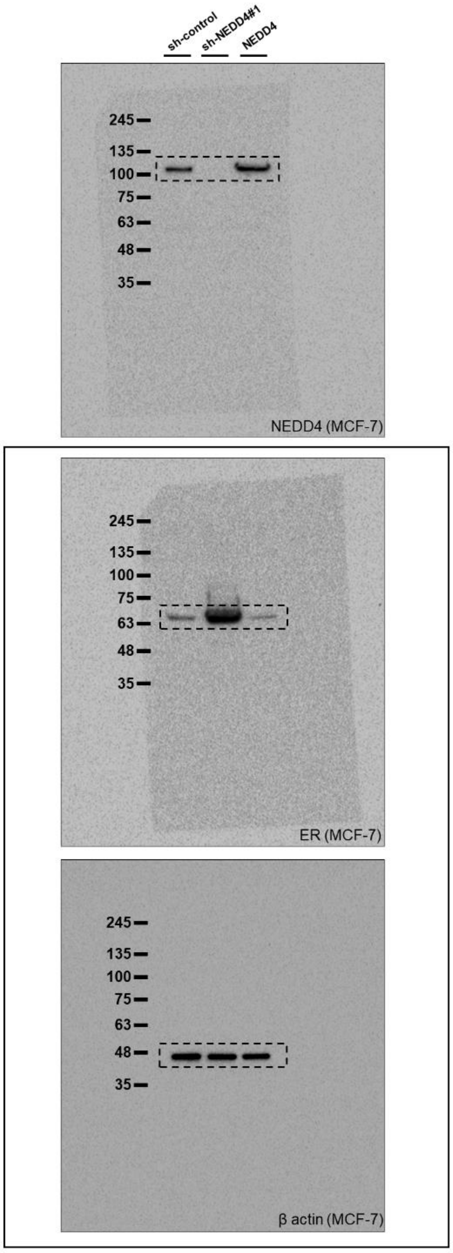

Figure S8. Full western blots of MCF-7 cell line for Figure 3C. Fields in the dotted line were cut out and used for the figure. Images of the same membrane that was re-blotted with different antibodies were grouped.

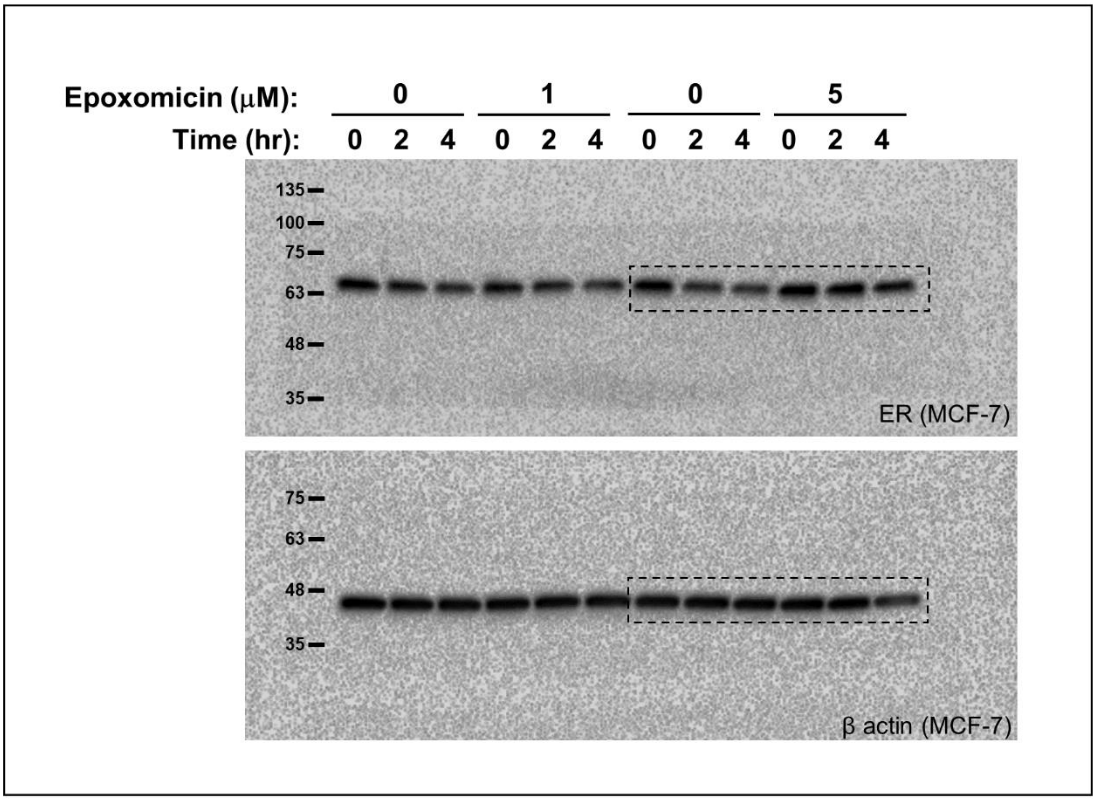

Figure S9. Full western blots of MCF-7 cell line for Figure 3D. Fields in the dotted line were cut out and used for the figure. Images of the same membrane that was re-blotted with different antibodies were grouped.

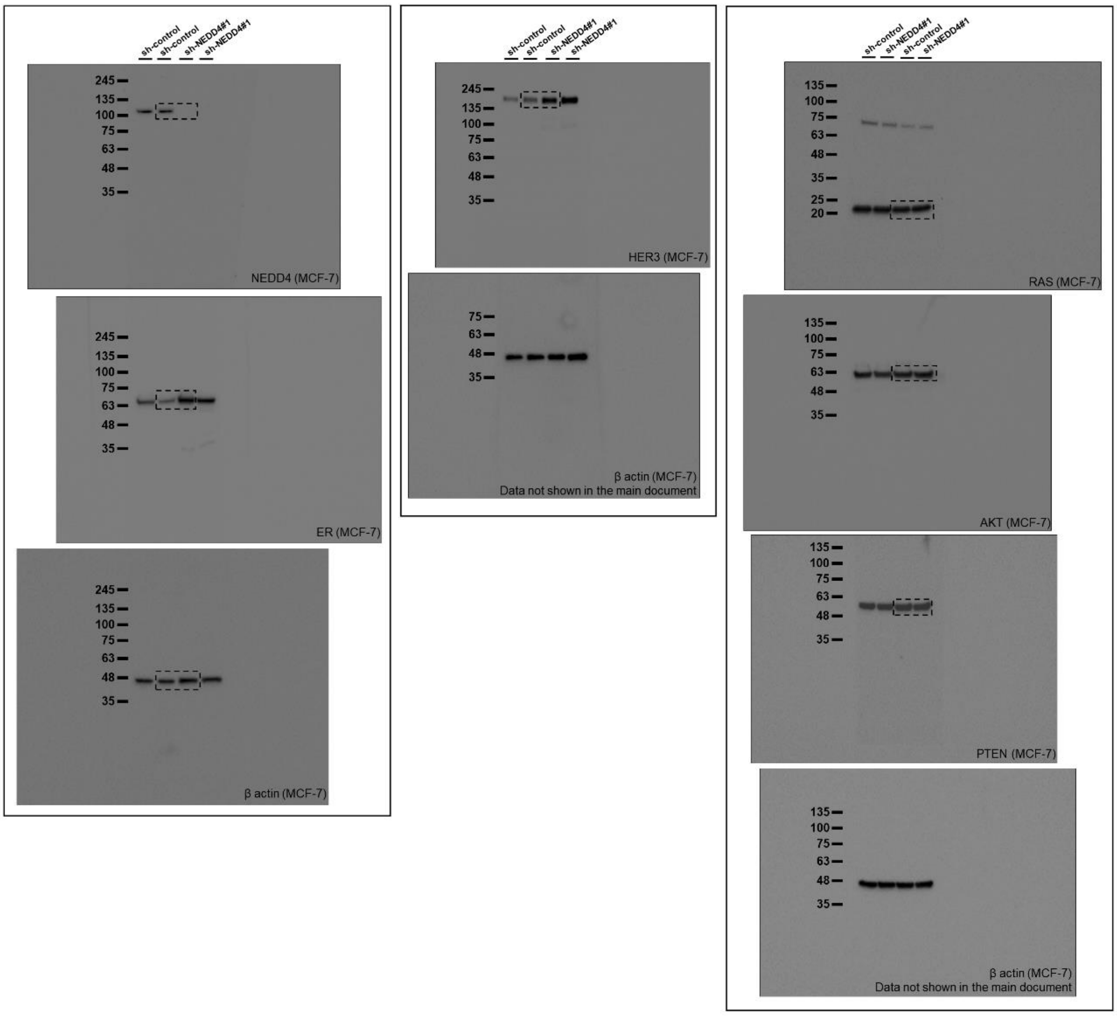

Figure S10. Full western blots of T47D cell line for Figure 3D. Fields in the dotted line were cut out and used for the figure. Images of the same membrane that was re-blotted with different antibodies were grouped.

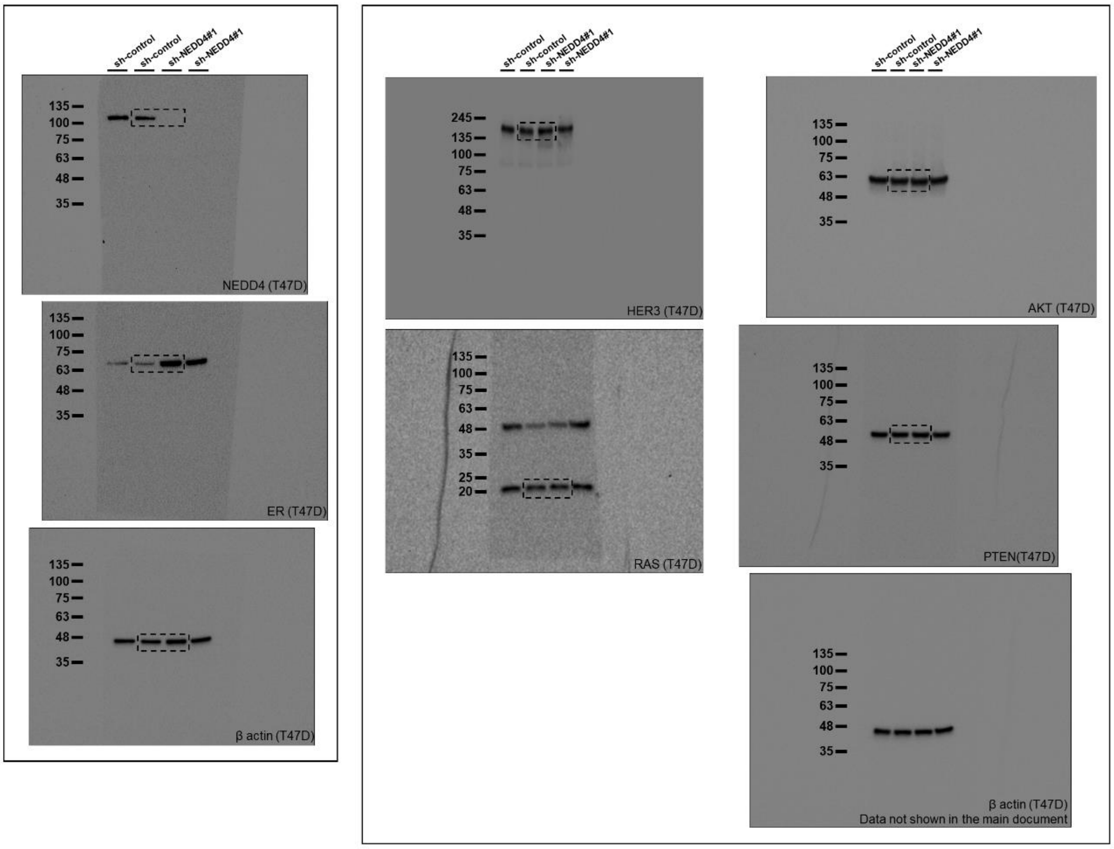

Figure S11. Full western blots of MCF-7 cell line for Figure 3E. Fields in the dotted line were cut out and used for the figure. Images of the same membrane that was re-blotted with different antibodies were grouped.

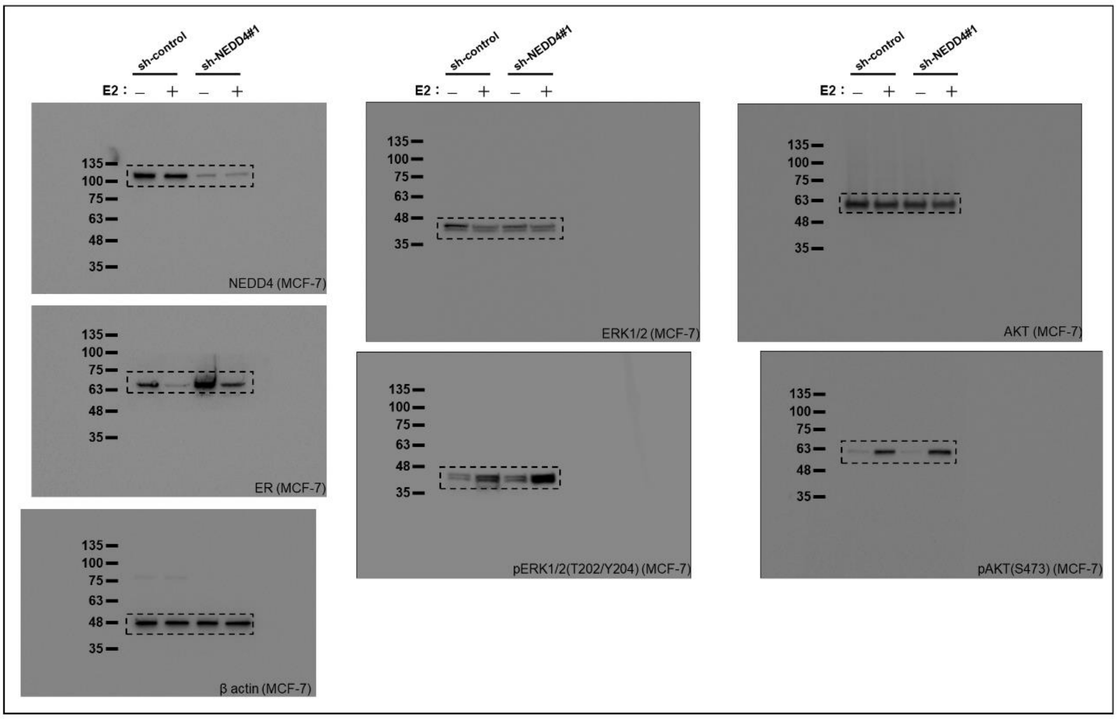

Figure S12. Full western blots of T47D cell line for Figure 3E. Fields in the dotted line were cut out and used for the figure. Images of the same membrane that was re-blotted with different antibodies were grouped.

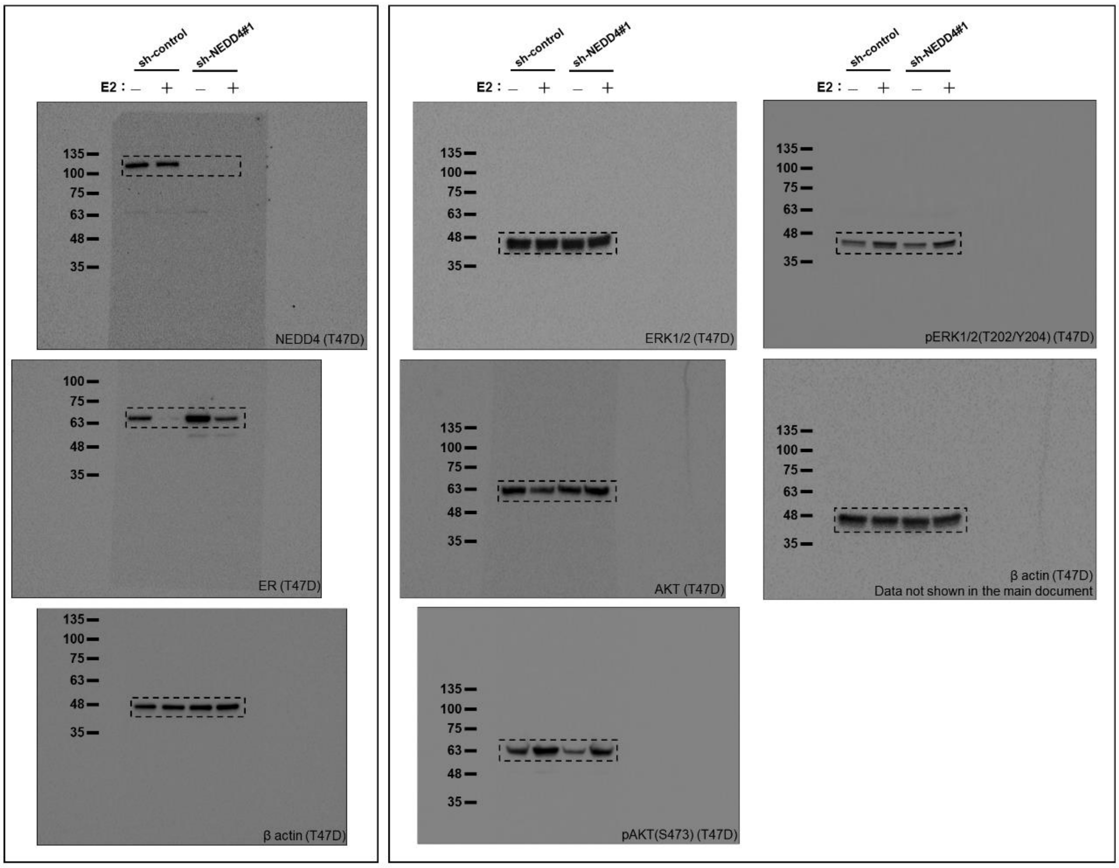

Figure S13. Bar charts based on the quantification of the Western blot bands of Figure 3E. Western blotting of sh-control and sh-NEDD4#1 MCF-7 cells stimulated with 1 nM E2 showed degradation of ER and phosphorylation of ERK and AKT. Bands of ER and β-actin on three independent images of the Western blot were quantified using densitometry. The three quantified intensities of bands were normalized by β-actin. The three relative intensities were averaged and made into a graph. The Western blot bands of phosphorylation of ERK1/2 (pERK1/2[T202/Y204]) and AKT (pAKT[S473]) were analyzed in the same way.

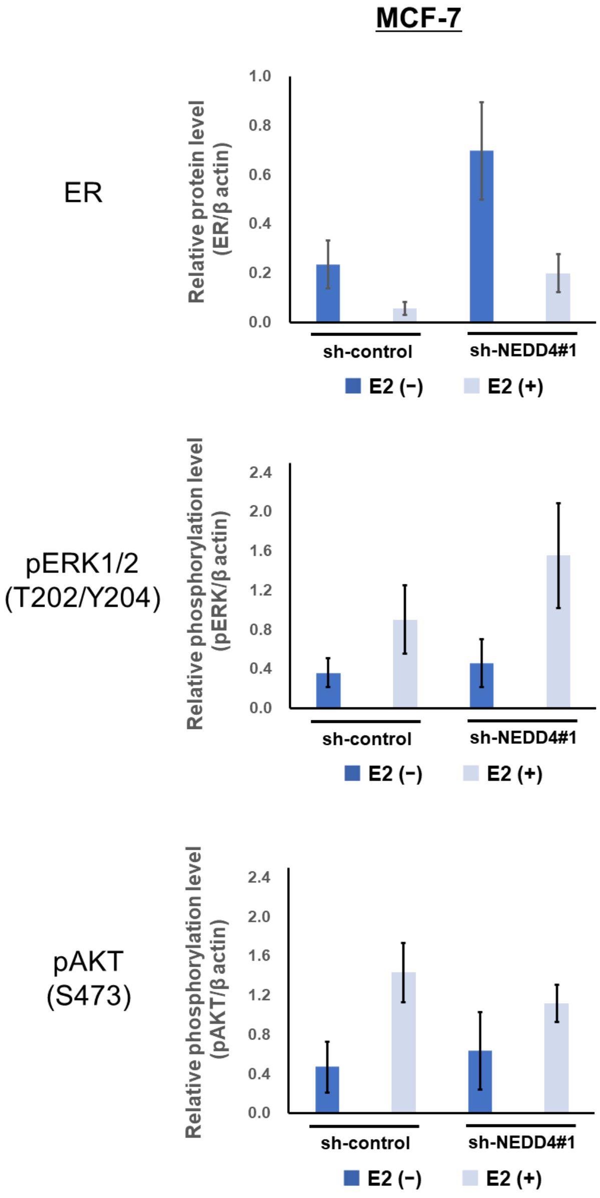

Figure S14. Full western blots of MCF-7 cell line for Figure 3F. Fields in the dotted line were cut out and used for the figure. Images of the same membrane that was re-blotted with different antibodies were grouped.

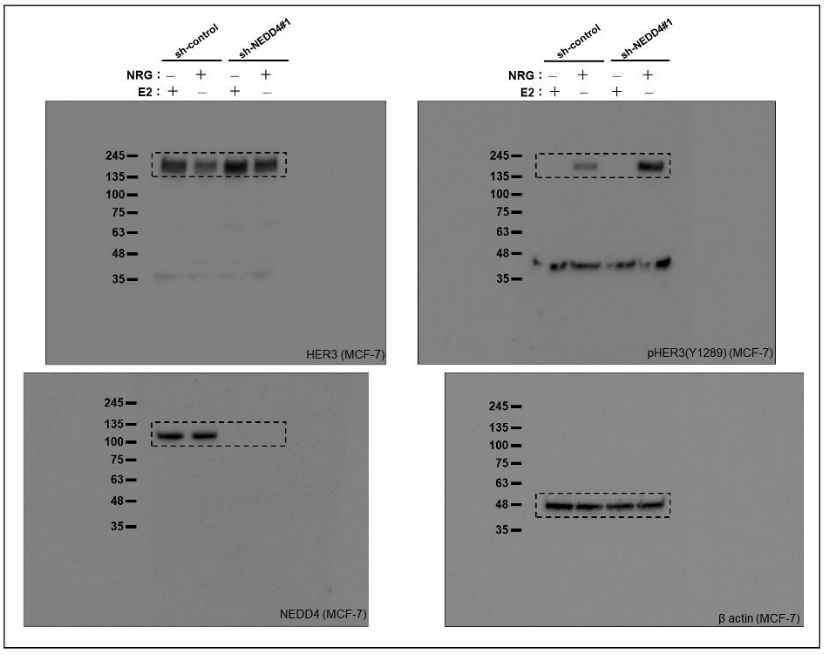

Table S1. The correlation between NEDD4 and ER expression level in clinical specimens. Of the 143 patients, 82 were divided into two groups at the NEDD4 mRNA level of -0.495. The percentage of positively ER-stained cells which were divided into four groups was compared.

	NEDD4 > -0.495 (n = 29)	NEDD4 < -0.495 (n = 53)	*P-value*
ER > 50% (%)	93.1	92.5	1.00
10% < ER < 50% (%)	6.9	5.7	1.00
1% < ER < 10% (%)	0.0	0.0	-
ER < 1% (%)	0.0	1.9	0.53
